# Effects of Olive (*Olea europaea* L.) Leaves with Antioxidant and Antimicrobial Activities on In Vitro Ruminal Fermentation and Methane Emission

**DOI:** 10.3390/ani11072008

**Published:** 2021-07-05

**Authors:** Shin Ja Lee, Hyun Sang Kim, Jun Sik Eom, You Young Choi, Seong Uk Jo, Gyo Moon Chu, Yookyung Lee, Jakyeom Seo, Kyoung Hoon Kim, Sung Sill Lee

**Affiliations:** 1 Institute of Agriculture and Life Science & University-Centered Labs, Gyeongsang National University, Jinju-si 52828, Gyeongsangnam-do, Korea; tlswk1000@hanmail.net; 2 Division of Applied Life Science (BK21), Gyeongsang National University, Jinju-si 52828, Gyeongsangnam-do, Korea; 2437401@naver.com (H.S.K.); skyandstar07@naver.com (J.S.E.); dudolboy301@naver.com (Y.Y.C.); jsu9412@naver.com (S.U.J.); 3 Nonghyupfeed INC. 337, Uam-ro, Nam-gu, Busan 48475, Korea; gyomoon96@hanmail.net; 4 Animal Nutrition and Physiology Team, National Institute of Animal Science, RDA, Jeonju-si 55365, Jeonrabuk-do, Korea; yoo3930@korea.kr; 5 Department of Animal Science, Life and Industry Convergence Research Institute, Pusan National University, Miryang 50463, Korea; jseo81@pusan.ac.kr; 6 Department of International Agricultural Technology, Graduate School of International Agricultural Technology, Seoul National University, Pyeongchang 25354, Gangwon-do, Korea; khhkim@snu.ac.kr; 7 Department of Ecofriendly Livestock Science, Institute of Green Bio Science and Technology, Seoul National University, Pyeongchang 25354, Gangwon-do, Korea

**Keywords:** olive leaves, antioxidant, antimicrobial agent, ruminal fermentation, methane production, animal feed

## Abstract

**Simple Summary:**

Olives are cultivated mostly in the Mediterranean as well as in Asia Minor, Korea, Japan, and China. Olive oil is currently used as a food ingredient in human diet, and its consumption is gradually expanding in various countries. Therefore, olive cultivation and oil extraction produce a significant amount of byproducts; providing these byproducts as feed to livestock has been attempted for a long period. Economic, environmental, and nutritional considerations make the use of olive byproducts efficient and cost-effective as feed for ruminants. Among the olive byproducts, olive leaves (OLs) contain higher levels of polyphenols than olive fruits, and have a very high feed value. In this study, it was confirmed that methane production decreased during 12 h of in vitro fermentation, and the number of fat-utilizing microorganisms increased in the 5% OLs group. OLs were found to show antioxidant and antimicrobial activity. Moreover, the proportion of cellulose-degrading bacteria, *Fibrobacter succinogenes*, *Ruminococcus albus*, and *Ruminococcus flavefaciens* increased in the 5% OLs group at 12 h and decreased at 24 h. Olive leaves are believed to be very useful as feed additives and supplements for ruminants.

**Abstract:**

We evaluated whether olive leaves (OLs) are effective as feed additives and supplements for ruminants and the potential methane reduction effects during in vitro fermentation. Two Hanwoo cows (460 ± 20 kg) equipped with cannula were fed Timothy hay and corn-based feed 3% of the body weight at a ratio of 6:4 (8:30 a.m. and 5:00 p.m.). Ruminal fluid from the cows was collected and mixed before morning feeding. In vitro batch fermentation was monitored after 12 and 24 h of incubation at 39 °C, and OLs were used as supplements to achieve the concentration of 5% in the basal diet. At 12 h of fermentation, methane production decreased in the 5% OLs group compared to that in the control group, but not at 24 h. The proportion of cellulose-degrading bacteria, *Fibrobacter succinogenes, Ruminococcus albus*, and *Ruminococcus flavefaciens*, tended to increase in the 5% OLs group at 12 h. The amount of ammonia produced was the same as the polymerase chain reaction result for *Prevotella ruminicola*. At 12 h, the proportion of *Prevotella ruminicola* was significantly higher in the 5% OLs group. OLs may be used incorporated with protein byproducts or other methane-reducing agents in animal feed.

## 1. Introduction

Olive leaves (OLs) are a byproduct of olive production and processing, accounting for up to 10% of the weight in olive production; their high polyphenol content (1%–14%) makes them an inexpensive source of antioxidant compounds [1,2]. They have been found to contain a large amount of high value-added antioxidants (oleuropein, hydroxytyrosol, carotene, triglycerides, tocopherol, and sitosterol) with antibacterial properties. Olive leaves (OLs) are used as feed additives and in medicine [3,4,5]. In particular, OLs are found to be effective in treating hypoglycemia and hypocholesterolemia due to their high antibacterial, antioxidant, and anti-inflammatory effects [6,7]. The antioxidant activity of OLs extracts (OLE) is mainly due to phenolic components including luteolin and hydroxytyrosol, as well as oleuropein, the main component in OLE, which have powerful anti-inflammatory, antibacterial, and antioxidant properties [8,9,10,11].

Olive leaves (OLs) have been used as animal feed due to their beneficial properties. Molina-Alcaide et al. [12] fed sheep and goats with olive tree byproducts and olive oil extract and studied the effects of OLs on digestion, decomposition, ruminal fermentation, animal performance, and product quality; they reported OLs to exert sufficient potential as feed for ruminants [12]. The most important greenhouse gas released from ruminal fermentation is methane (CH_4_). In the study of Shakeri et al. [13], OLs and chloroform OLE reduced CH_4_ production by reducing ammonia (NH_3_) production and increasing propionate level in the rumen, suggesting that they could help reduce CH_4_ production when supplemented in feed. The fatty acid composition in OLs byproducts is a particularly important factor. In addition, OLs are characterized by low digestibility and low crude protein (CP) content [14]. However, if properly supplemented, it can be successfully used in animal feed [15].

The use of olive cakes in ruminant feeds results in different rates of ruminant fermentation and digestibility depending on the method of administration and the proportion in the diet [16]. Based on the results of evaluation of apparent digestibility and digestibility in the intestine, olive cakes can be used in silage or feed blocks [12].

Extracted olive cakes provide cheap energy and fiber to animals, and high-fat olive cakes can be used to improve the fat quality of animal products [12]. Olive leaves (OLs) represent a valuable material for developing functional animal feed. In this study, we determined whether antioxidant-rich OLs have potential as CH_4_ reducing feed additives.

## 2. Materials and Methods

### 2.1. Sample Preparation

Olive leaves (OLs) were obtained from Spain (Teetraum, Wollenhaupt Co., Ltd., Reinbek, Germany), and were purchased from CJ ENM Co., Ltd. (Seocho-gu, Seoul, Korea). Olive leaves (OLs) were dried for 2 h in a dry oven (TEIOTECH, Daejeon, Korea) at 55 °C and crushed with a 2 mm screen using a Wiley mill (Model 4; Thomas Scientific, Swedesboro, NJ, USA). Analysis of the general components of OLs was performed according to the AOAC method [17]; the following components were analyzed: dry matter (DM; method 934.01), CP (method 954.01), crude fiber (CF; method 962.09), ether extract (EE; method 920.39), and crude ash (CA; method 942.05). Neutral detergent fibers (NDF) were analyzed using heat-resistant amylase, and acid detergent fibers (ADF), including residual ash, were analyzed using the method of Van Soest, Robertson, and Lewis [18].

### 2.2. In Vitro Batch Fermentation

Two rumen-fistulated, non-lactating Hanwoo cows of body weight 460 ± 20 kg were used as ruminal inoculum donors. They were fed a basal diet of 60% Timothy hay and 40% corn-based feed (CP, 120 g/kg; EE, 15 g/kg; CF, 150 g/kg; CA, 120 g/kg; Ca, 7.5 g/kg; P, 9.0 g/kg; 690 g/kg DM basis) at the energy maintenance level (3% DM of their BW). The cows had free access to clean drinking water and a mineral block. Feed was offered at 8:30 a.m. and 5:00 p.m. Ruminal fluid was collected and mixed before morning feeding from two Hanwoo cows. After passing through it using 4 layers of cheese cloth, it was placed in an insulated bottle maintained at 39 °C and moved to the laboratory in a vacuum state [19]. Rumen fluid was mixed with McDougall’s buffer [20] artificial saliva in a ratio of 1:2 (V:V). A 15 mL mixing buffer was added to a 50 mL serum bottle, and 300 mg (based on DM) of Timothy as a substrate was prepared (control: without OLs, 0%; 5% OLs group: supplementation 5% of OLs at the rate of Timothy hay). All experiments were conducted by dispensing carbon dioxide (CO_2_) gas to maintain anaerobicity, and the serum bottle was sealed with a butyl rubber stopper and an aluminum cap to maintain anaerobicity. The experimental design was completely randomized and was run in triplicate (*n* = 3). Gas production was monitored after 12 and 24 h of incubation at 39 °C.

### 2.3. Total Polyphenols, Total Flavonoid Contents, and Antioxidant Activity

Total phenol content was analyzed by the method of Singleton and Rossi [21]. The analytical procedure was to react 0.5 mL of diluted sample with 2.5 mL of 0.2 M/L Folin-Ciocalteu reagent for 4 min, and 2 mL of saturated sodium carbonate solution (75 g/L) was added to the reaction mixture. After incubation at room temperature for 2 h, absorbance measurements were measured at 760 nm. Gallic acid (GAE) was used as a reference standard calibration curve and the results were expressed as milligram gallic acid equivalent (mg GAE)/g dry weight (g DW).

Total flavonoids were used by modifying the method of Meda et al. [22]. A sample (0.25 mL) was placed in a tube containing 1 mL of double-distilled water, 5% NaNO_2_ was added, and after 5 min, 0.075 mL of 10% AlCl_3_ was added to react, and 0.5 mL of 1 M NaOH was added after 1 min. The volume of the reaction solution was adjusted to 2.5 mL with double-distilled water. The absorbance of the solution at a wavelength of 410 nm was measured using a spectrophotometer (Ultrospec 2100 pro, Amersham Pharmacia Biotech Co., Piscataway, NJ, USA). Quercetin was used as a standard calibration curve to quantify total flavonoid content. Results were expressed as milligram quercetin equivalents (mg QE)/g dry weight (g DW).

Sample treatment for analyzing 2,2-diphenyl-1-picrylhydrazyl (DPPH) radical scavenging activity (0.05–1 mg/mL in DMSO) was used by modifying the method of Brand-Williams et al. [23] to suit the experimental purpose. The standard material for radical scavenging activity was used as L-ascorbic acid. The radical scavenging activity of the sample was calculated and expressed by the IC50 value.

2,2′-Azino-bis(3-ethylbenzothiazoline-6-sulfonic acid (ABTS) radical scavenging activity was determined using the method reported by Re et al. [24]. 2,2′-Azino-bis(3-ethylbenzothiazoline-6-sulfonic acid (7 mM) and potassium sulfate (2.45 mM) were reacted at room temperature for 12 h to form an ABTS radical cation (ABTS +). The ABTS + solution was measured by absorbance at 734 nm. The radical scavenging activity of the tested samples was calculated and expressed as the IC50 value.

The hydroxyl (HO) radical scavenging activity of OLE at various concentrations was determined by Elizabeth et al. [25]. The absorbance of the mixture was measured at 532 nm. Standard substances of HO radical scavenging activity were compared and analyzed using butylated hydroxyanisole (BHA) and catechin.

Nitric oxide (NO) radical scavenging ability was measured by modifying the Lee [26] method. The modified content was analyzed by the Griess-Ilosvay reaction. Butylated hydroxyanisole (BHA) was used as the standard, and absorbance was measured at 546 nm.

### 2.4. Assessment of Antimicrobial Assay

Antibacterial analysis was performed using a standard disc diffusion method using *Escherichia coli, Pseudomonas aeruginosa, Klebsiella pneumoniae*, and *Staphylococcus aureus*. McFarland standard culture conditions were used after incubation at 37 °C for 24 h. Saturated cultures were taken, inoculated on Mueller Hinton agar plates, and bacteria were cultured to determine the vitality of antibacterial assays. For OLE, 50 μL of each plate was placed on a sterile paper disc with a diameter of 5 mm, incubated at 37 °C for 24 h, and the surrounding clear zone was checked to measure the antimicrobial activity based on the inhibitory area [27,28].

As for the criteria for determining the clear zone, when the medium was visually checked at a distance of about 30 cm, the range in which bacteria did not grow at all was recognized as the clear zone. If the clear zone was elliptical, the smaller diameter was recognized as the clear zone. The areas where the bacteria grew lightly or had single colonies were excluded from the clear zone [28].

### 2.5. Analysis of Metabolites in Gas Chromatography-Mass Spectrometry (GC-MS)

Gas chromatography-mass spectrometry (GC-MS) was used to analyze carbohydrate metabolites in OLE. For the analysis, an RTx-5MS capillary column (30 m × 0.25 mm, id × 0.25 μm film thickness, Restek Co., Bellefonte, PA, USA) was used. As for the analysis conditions, helium gas was used as a transport gas at a flow rate of 0.95 mL/min, and the initial 2 min was increased from 70 °C to 300 °C at a rate of 20 °C/min. It was kept in this state for 3 min. The transfer line temperature was 280 °C and the ion source temperature was 230 °C. The scans event time source was 0.003 s, 15 eV scan. MS data ranged from *m*/*z* 45~550 [29].

### 2.6. Analysis of In Vitro Fermentation

The supernatant of the culture medium was sampled to analyze pH, volatile fatty acid (VFAs), and microbial growth rate (MRG). The pH was measured using a pH meter (MP230, Mettler-Toledo, Greifensee, Switzerland). To analyze VFAs (total VFA, acetate, propionate, and butyrate), the supernatant of the culture medium was centrifuged at 10,483× *g* for 3 min, and the supernatant was collected and used for analysis. The content of VFAs was measured by high-performance liquid chromatography (L-2200; Hitachi, Tokyo, Japan). Their content was individually calculated using the standard curve equation, and the value of ppm was converted to mmol/L. Microbial growth rate (MRG) was determined by centrifuging the supernatant at 655× *g* for 3 min, then centrifuging once more at 14,269× *g*, washing 4 times with sodium phosphate buffer and then using a spectrophotometer (Model 680, Bio-Rad Laboratories, Hercules, CA, USA) to analyze the OD (optical density) value at 550 nm.

Total gas production was measured using a digital readout voltmeter (Laurel Electronics, Inc., Costa Mesa, CA, USA) in the headspace above the culture bottle during each fermentation time. The pressure of the gas was recorded on the LED display after the hypodermic needle was inserted into the culture bottle. Gas samples for CH_4_ and CO_2_ analysis were transferred to vacuum test tubes (Vacutainer, Becton Dickinson, Franklin Lakes, NJ, USA) and analyzed using a column TCD detector (HP 5890; Agilent Technologies, Santa Clara, CA, USA). Carboxen 1006PLOT capillary column 30 m × 0.53 mm (Supelco) thermal conductivity detector was used [30].

### 2.7. Total DNA Extraction and Quantitative Real-Time Polymerase Chain Reaction

Total DNA was extracted from the pellet stored at −80 °C using the repeated bead beating in the subsequent DNA purification by QIA^®^ columns (referred to as repeated bead beating plus column (RBB + C) method) [31]. Genomic DNA was treated with RNase A and proteinase K and purified using columns from the DokDo-Prep Genomic DNA Kit (Elpis-Biotech, Daejeon, Korea). The concentration and purity of total DNA were measured using a NanoDrop (ND-1000, Thermo Fisher, Waltham, MA, USA). Quantitative real-time polymerase chain reaction (PCR) assays were performed on a CFX 96 Touch system (Bio-Rad Laboratories, Inc., Hercules, CA, USA). Quantitative real-time PCR was carried out according to the manufacturer’s instructions, as follows.

The initiation for one cycle at 95 °C for 10 min, 40 cycles each for denaturation at 95 °C for 30 s, annealing at 60 °C for 30 s, and elongation at 72 °C for 30 s, and final elongation at 72 °C for 5 min.

Fluorescence was recorded at the end of each denaturation and extension step, and the specificity of the sample was confirmed via dissociation curve analysis of PCR end products by increasing the temperature at a rate of 1 °C every 30 s, from 60 °C to 95 °C.

The PCR primers include general bacteria [32], ciliated protozoa [33], fungi [32], methanogenic archaea [33], *Fibrobacter succinogenes* (*F. succinogenes*) [32], *Ruminococcus albus* (*R. albus*) [34], *Ruminococcus flavefaciens* (*R. flavefaciens*) [32], *Prevotella ruminicola* (*P. ruminicola*) [33], *Butyrivibrio fibrisolvens* (*B. fibrisolvens*) [35], *Butyrivibrio proteoclasticus* (*B. proteoclasticus*) [36], and *Anearovibrio lipolytica* (*A. lipolytica*) [37] (Table 1).

For absolute quantification of each microbe, PCR cloning was performed using each of the primers described in Table 1 to obtain a standard plasmid containing each target gene sequence.

The copy number of each standard primer was calculated [38]. If the molecular weight of the plasmid and insert is known, it is possible to calculate the copy number as follows:

Weight in daltons (g/mol) = (bp size of ds product)(330 Da × 2 nt/bp)

Hence: (g/mol)/Avogadro’s number = g/molecule = copy number

where: bp = base pairs, ds = double-stranded, nt = nucleotides.

CFX manager software (Bio-Rad, USA) was used to compare microbe quantifications with the standard curve.

### 2.8. Statistical Analysis

The effects of antioxidants and nitric oxide were decomposed into three orthogonal polynomial contrasts (linear, quadratic and cubic). Coefficients were generated using the IML procedure in Statistical Analysis System (SAS 9.4 Institute Inc., Cary, NC, USA) [39]. Duncan’s multiple comparison test was used to investigate significant differences between the sample means. An independent-samples *t*-test was conducted to test the significance of differences in the effects of OLs on the in vitro rumen fermentation, rumen microbial populations, and CH_4_ production. Results with a *p* value of < 0.05 were considered statistically significant, and results with a 0.05 ≤ *p* value < 0.1 were considered to have tendency.

## 3. Results

### 3.1. Chemical Composition of OLs and Their Total Phenolic and Flavonoid Contents

To determine the quality of the OLs used in the study, we analyzed their chemical composition. The mean values of the chemical components are presented in Table 2. The mean values of the different components are expressed as the mean ± standard deviation. The components of DM, CP, and EE were 94.59% ± 0.03%, 8.8% ± 0.30%, and 10.87% ± 0.49%, respectively. The CA content was 8.28 ± 0.17%, NDF and ADF contents were 38.82 ± 0.81% and 27.35 ± 0.11%, respectively.

The total phenolic and total flavonoid content in OLs was 34.79 ± 2.72 mg catechin/g and 5.91 ± 0.24 mg quercetin/g, respectively (Table 2). The IC50 of 2,2-diphenyl-1-picrylhydrazyl (DPPH) and 2,2′-azino-bis(3-ethylbenzothiazoline-6-sulfonic acid (ABTS) of the standard was 78.14 and 33.21 μg/mL, respectively. Olive leaves (OLs) tended to show a relatively higher antioxidant activity at high concentrations (10, 50, 100, and 200 μg/mL). All homeostatic activities were highest at a concentration of 200 μg/mL. The hydroxyl (HO) radical-scavenging activity was high compared to other scavenging activities (Table 3). The antioxidant and NO radical inhibition effect of the extract at 200 μg/mL concentration was detected using the DPPH (54.25%), ABTS (92.36%), HO (596.4%), and NO (27.94%) assays.

### 3.2. Carbohydrate Composition in OLs

Olive leaves (OLs) contained sucrose (39.71%) as the dominant carbohydrate, followed by sorbitol (26.30%) and glucose (10.42%). In this analysis, 62% of the metabolites identified as a whole were carbohydrate classes (CHO) (Table 4).

### 3.3. In Vitro Batch Fermentation

The pH of OLs ranged from 6.61 to 7.28. The pH at 12 h was 7.11 and 7.28 in the control and 5% OLs groups, respectively; the pH was 6.61 and 6.66 in the control and 5% OLs groups at 24 h, respectively.

There was no significant difference in the MGR between the 5% OLs group and control group at 24 h (*p* > 0.05).

Dry matter digestion was affected by feed supplemented with 5% OLs after 12 and 24 h of incubation (*p* < 0.05). Dry matter degradation after 12 and 24 h was significantly reduced in the 5% OLs groups.

The concentration of ammonia (NH_3_) was significantly increased in the 5% OLs group (*p* < 0.05).

Olive leaves (OLs) showed similar molar percentages of acetate and propionate at 12 and 24 h. The acetate-to-propionate (A/P) ratio was high in the control group at 12 h and significantly higher in (*p* = 0.0029) the 5% OLs group at 24 h. Moreover, the total VFA (*p* = 0.0023) and acetate (*p* = 0.0002) content in the 5% OLs group was higher than that in the control group at 12 h, and there was no significant difference in the propionate content between the groups (*p* > 0.05) (Table 5).

The amount of CH_4_ produced based on the amount of total VFA produced was determined, and it was confirmed that CH_4_ production was reduced in the OLs group at 12 h. This result was consistent with the amount of CH_4_ produced via in vitro batch fermentation.

**Figure 1:**
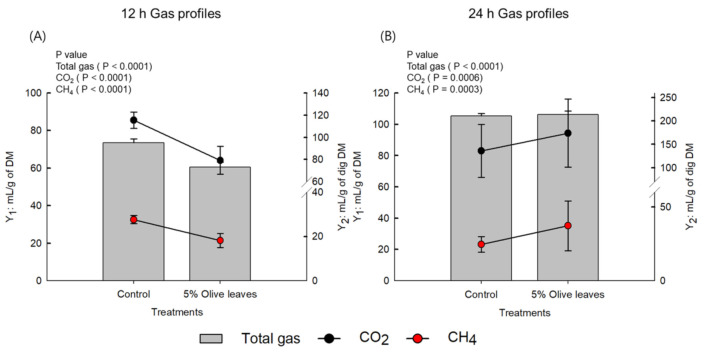
Effects of 5% olive leaves (OLs) on gas profile at (**A**) 12 and (**B**) 24 h. Y_1_ axis represents gas production unit (mL/g of DM) and Y_2_ axis represents gas production unit (mL/g of dig DM). Error bars indicate standard error of the mean (*n* = 3).

Methane (CH_4_) emission at 12 h was significantly lower in the 5% OLs group (18.19 mL/g dig DM, *p* < 0.0001) than in the control group (27.63 mL/g dig DM, *p* = 0.0006). The total gas and CO_2_ emissions were similar to the pattern of CH_4_ emission, and were low in the 5% OLs group (Figure 1).

### 3.4. Antibacterial Activity

Antibacterial activity was measured against *Staphylococcus aureus, Escherichia coli, Klebsiella pneumoniae,* and *Pseudomonas aeruginosa* via paper disc diffusion assays; the results are shown in Table 6 and Figure S1. The 5% OLs fraction showed the highest activity against *S. aureus*. These findings confirmed that OLs had antibacterial activity against pathogenic bacteria.

### 3.5. Effect of OLs on the Microbial Composition on In Vitro Batch Fermentation

The absolute value of total bacteria in the 5% OLs group was significantly lower than control group (Table 7). Olive leaves (OLs) supplementation reduced the total bacteria at both 12 and 24 h. The abundance of fungi decreased in the 5% OLs group at 24 h, and the abundance of ciliate protozoa was unaffected via OLs group. The absolute value of methanogenic archaea did not differ at 12 h, and was significantly lower at 24 h (*p* < 0.0001). The relative proportions of fibrolytic bacteria including *R. albus* and *R. flavefaciens* and that of *P. ruminicola* tended to increase at 12 h in the 5% OLs group compared to controls. However, most microorganisms showed the opposite trend at 24 h compared to that observed at 12 h. The absolute values of *F. succinogenes* and *R. albus* were high in the 24 h control group (*p* < 0.0001). There was no significant difference in the proportion of *R. flavefaciens* at 24 h. The abundance of proteolytic bacteria including *B. fibrisolvens* and *B. proteoclasticus* was significantly lower in the 5% OLs group than in the control group. In addition, the relative proportion of fat-utilizing bacteria, *A. lipolytica*, was significantly higher in the 5% OLs group (*p* < 0.0001).

## 4. Discussion

The chemical composition of OLs was investigated. According to Molina-Alcaide et al., the chemical composition in OLs (g/kg DM) comprises DM (777), EE (56.4), CP (100), mild detergent fiber (406), and ADF (302). In this study, the composition (g/kg DM) included DM (945.9), CP (108.7), EE (88), NDF (388), and ADF (273.5), which was similar to that reported in literature.

Olive leaves extract (OLE) has been found to contain antibacterial and antioxidant properties [40]. Brahmi et al. [41] suggested that the antibacterial activity of OLs is related to the terpene content. In this study, the terpene-based polyphenol content was found to be 34.79 mg catechin/g and the flavonoid content was 5.91 mg quercetin/g. Terpenes are known to disrupt cell membranes [42]. Olive leaves extract (OLE) has been shown to regulate the composition of gastric bacteria by selectively reducing the levels of *Campylobacter jejuni* and *Helicobacter pylori* [43]. Although many studies have investigated the antibacterial activity of OLs, the mechanism of action of the antimicrobial activity has not been fully elucidated [40]. Olive leaves extract (OLE) has been found to show antibacterial activity against *S. aureus*, *E. coli*, and *Salmonella* spp.; a particularly high antimicrobial activity is observed against *Listeria monocytogenes* [44].

We found that digestibility in the in vitro batch was significantly lower in the OLs group than in the control group. According to Molina-Alcaide et al. [11], information on rumen CP decomposition after OLs supplementation is scarce, and the reported values are low and the variance is large. Olive leaves (OLs) and olive cake show low ruminal decomposition in both sheep and goats. Moreover, the addition of polyethylene glycol to olive byproducts increases rumen-derived microbial nitrogen, fermentation properties, and the degradability of DM [11].

Olive leaves extract (OLE) using chloroform has various physiological activities, and contains a large amount of secondary compounds, such as phenolic compounds and condensed tannins. It is also effective in reducing CH_4_ production without affecting the VFA concentration [13]. Shakeri et al. [13] reported that the addition of OLs to in vitro fermentation reduces CH_4_ emissions in the rumen by 15–53% compared to that in the control. Olive leaves (OLs) group significantly reduces the A/P ratio by increasing propionate production, which activates the pathway for reducing CH_4_ production. Molina-Alcaide et al. [10] tested continuous culture fermenters and reported that the A/P ratio is lower when using diet containing OLs than that obtained using alfalfa hay. The decrease in the A/P ratio via OLs addition may be due to the reduced growth of ruminant cellulolytic bacteria owing to antibacterial activity.

The reason why OLs are associated with a lower gas production and total VFA concentration than those obtained using the control may be attributed to their low nutritional value [11]. Phenolic compounds in OLs are known to affect fermentation. In addition, these phenolic compounds show antimicrobial effects against ruminant microbes, thereby reducing the overall microbial activity in a similar way [45,46]. In this study, we found that the total VFA decreased, and the phenolic compounds present in OLs interfered with the activity of ruminant microorganisms. The 80% ethanol extract, butanol, and ethyl acetate fractions of OLs inhibited the growth of *Bacillus cereus*, *S. aureus*, *E. coli*, and *Salmonella enteritidis*; however, the hexane and chloroform fractions did not show antibacterial activity. OLs at concentrations of <100 µg/disc do not exert antibacterial effects against *Bacillus cereus*, *S. aureus*, *E. coli*, and *Salmonella enteritidis*, and at the concentration of 400–800 µg/disc, the size of clear zones of inhibition is 11–20 mm [47].

The bacterial inhibition activity was assessed based on the clear zone diameter as follows: very strong (>20 mm), strong (10–20 mm), medium (5–10 mm), and weak (<5 mm) [48]. In this study, zones of inhibition of sizes 8–10 mm were obtained when bacteria were inoculated with 50 μg of OLs group. The antibacterial effect increased with an increase in concentration of OLs group. We detected antibacterial activity despite a low concentration. Lee et al. [47] suggested that the butanol and ethyl acetate fractions containing high contents of flavonoids, total phenols, and phenolic compounds show potent antimicrobial activity, suggesting that there is a correlation between antibacterial activity and phenolic substances. The antimicrobial activity of OLs is often associated with its major phenolic component, alleuropein [49]. Lee-Huang et al. [49] reported that phenolic compounds such as rutin, verbascoside, luteolin-7-glucoside, apigenin-7-glucoside, oleuropein, and oleuroside are present in OLs, and that only oleropein and oleuroside are capable of anti-HIV activity. In addition, the results of antimicrobial activity are also linked to a decrease in digestibility, which may be due to a high fat content and a large amount of phenolic compounds, resulting in a decrease in digestibility, thereby decreasing the total gas emission. However, these naturally derived substances do not have long-lasting and potent antioxidant and antimicrobial effects. Therefore, the growth of microorganisms and the amount of gas generated may have increased within 24 h [50]. At 12 h of fermentation, the production of CH_4_ decreased (*p* < 0.0001) compared to the control in the 5% OLs group, but not at 24 h. Therefore, the effect of OLs slowing down CH_4_ production was not long-lasting. The addition of plant-derived oils to feed can affect ruminant microorganisms. Ernesto Vargas et al. [51] showed that the total bacterial count was significantly reduced. This is consistent with reports that OLs has antibacterial properties [43]. This is an important result of examining the antibacterial mechanism of OLs against ruminal fluid. The total bacterial count decreased at both 12 and 24 h in the OLs group (*p* = 0.021).

The abundance of fungi decreased 8.4-fold in the OLs group at 24 h. Several studies have reported that a high-fat diet reduces the population of protozoa; however, there was no significant difference in this study. The proportion of protozoa in the rumen of OLs-fed animals is lower than that in standard diet-fed animals, possibly due to a lack of water-soluble carbohydrates and a high content of unsaturated fatty acids present in OLs. However, supplementation of OLs with barley grains or faba beans is associated with adequate microbial activity in goats and sheep [15].

The proportion of methanogenic archaea in the OLs group was 2.8-fold lower than that in the control group at 24 h. The reason CH_4_ emission did not decrease at 24 h despite the decrease in the proportion of methanogenic archaea may be partly due to the relative abundance of CH_4_-producing archaea in the rumen [52].

The proportion of cellulose-degrading bacteria, *F. succinogenes, R. albus*, and *R. flavefaciens*, tended to increase in the OLs group at 12 h and decreased at 24 h. *P. ruminicola* is known to degrade hemicellulose and produce NH_3_ [53]. In this study, the amount of NH_3_ produced was the same as the microbial composition result for *P. ruminicola*. At 12 h, the proportion of *P. ruminicola* was significantly higher in the 5% OLs group than that in the control group. The amount of NH_3_ produced increased over 12 h. In addition, even with the decrease in *P. ruminicola* abundance at 24 h, NH_3_ levels were high in the 5% OLs group. The content of NH_3_ was higher in the 5% OLs group than in the control. In the 5% OLs group, there was no difference in the NH_3_ content with time, with values of 13.49 and 13.02 mg∙dL^−1^ at 12 and 24 h, respectively. Presumably, *P. ruminicola* abundance was higher in the 12 h 5% OLs group than in the control group, where OLs addition had more protein sources than the control group.

The proportion of proteolytic bacteria, *B. fibrisolvens* and *B. proteoclasticus*, was found to be low in the 5% OLs group, which may be attributed to the low protein content in OLs. When OLs with relatively high fatty acid content were used, the abundance of lipolytic bacteria such as *A. lipolytica* was significantly higher in the 5% OLs group. Olive leaves (OLs) may be fully utilized as a high-energy feed by supplementing protein and feeding OLs at an appropriate ratio.

## 5. Conclusions

Several byproducts of olives have been identified and studied; however, the byproducts related to OLs have not been studied for their use as feed for livestock. Olive leaves (OLs) have a higher polyphenol content than that in olives, and when byproducts are provided as feed, they exhibit antibacterial effects against pathogenic bacteria and antioxidant effects in animals. In this study, it was confirmed that CH_4_ emission decreased during 12 h of in vitro fermentation, and the number of fat-utilizing microorganisms increased in the 5% OLs group. Consumption of these byproducts, during the currently faced issues of climate change, may present a viable strategy. In addition, OLs are considered as good feed additives for ruminants when supplemented with proteins. Future studies should evaluate supplements comprising OLs and proteins.

## Figures and Tables

**Table 1:** Polymerase chain reaction primer for quantitative real-time polymerase chain reaction assays.

Target Species	Primer	Sequence (5′ → 3′)	Size (bp)	Reference
General bacteria	F	CGGCAACGAGCGCAACCC	130	[32]
	R	CCATTGTAGCACGTGTGTAGCC		
Ciliate protozoa	F	GCTTTCGWTGGTAGTGTATT	223	[33]
	R	CTTGCCCTCYAATCGTWCT		
Fungi	F	GAGGAAGTAAAAGTCGTAACAAGGTTTC	120	[32]
	R	CAAATTCACAAAGGGTAGGATGATT		
Methanogenic archaea	F	GAGGAAGGAGTGGACGACGGTA	232	[33]
	R	ACGGGCGGTGTGTGCAAG		
*Fibrobacter succinogenes*	F	GTTCGGAATTACTGGGCGTAAA	121	[32]
	R	CGCCTGCCCCTGAACTATC		
*Ruminococcus albus*	F	CCCTAAAAGCAGTCTTAGTTCG	176	[34]
	R	CCTCCTTGCGGTTAGAACA		
*Ruminococcus flavefaciens*	F	CGAACGGAGATAATTTGAGTTTACTTAGG	132	[32]
	R	CGGTCTCTGTATGTTATGAGGTATTACC		
*Prevotella ruminicola*	F	GCGAAAGTCGGATTAATGCTCTATG	78	[33]
	R	CCCATCCTATAGCGGTAAACCTTTG		
*Butyrivibrio fibrisolvens*	F	ACCGCATAAGCGCACGGA	65	[35]
	R	CGGGTCCATCTTGTACCGATAAAT		
*Butyrivibrio proteoclasticus*	F	TCCGGTGGTATGAGATGGGC	185	[36]
	R	GTCGCTGCATCAGAGTTTCCT		
*Anearovibrio lipolytica*	F	TGGGTGTTAGAAATGGATTC	597	[37]
	R	CTCTCCTGCACTCAAGAATT		

**Table 2:** Chemical composition and antioxidant activity of olive leaves (OLs) (DM basis, %).

Items	*Olive leaves* (Mean ± SEM)
**Chemical composition**	
Dry matter (DM)	94.59 ± 0.03
Crude protein	10.87 ± 0.49
Ether extract	8.80 ± 0.30
Crude ash	8.28 ± 0.17
Neutral detergent fiber	38.82 ± 0.81
Acid detergent fiber	27.35 ± 0.11
**Antioxidant activity**	
Total polyphenol (mg catechin/g extract)	34.79 ± 2.72
Total flavonoid (mg quercetin/g extract)	5.91 ± 0.24
IC50 for DPPH (μg/mL) ^1^	78.14
IC50 for ABTS (μg/mL) ^2^	33.21

^1^ IC50: DPPH: 2, 2-Diphenyl-1-picrylhydrazyl radical scavenging activity; ^2^ ABTS: 2, 2’-Azino-bis (3-ethylbenzothiazoline-6-sulfonic acid) radical scavenging activity. SEM: standard error of the mean

**Table 3:** Antioxidant capacities (DPPH, ABTS, HO) and nitric oxide (NO) inhibition of olive leaves (OLs) (DM basis, %).

Content ^1^	Plant Concentration (µg/mL, Mean ± SEM)				SEM ^2^	*p*-Value	Contrast ^3^		
	10	50	100	200			L	Q	C
DPPH	33.18 ± 1.52 ^c^	47.37 ± 1.15 ^b^	53.21 ± 0.81 ^a^	54.25 ± 0.43 ^a^	1.06	<0.0001	<0.0001	<0.0001	0.0434
ABTS	82.40 ± 0.94 ^d^	87.84 ± 0.24 ^c^	90.80 ± 0.09 ^b^	92.36 ± 0.11 ^a^	0.38	<0.0001	<0.0001	<0.0001	0.0859
HO	63.62 ± 0.47 ^d^	104.15 ± 0.12 ^c^	265.78 ± 0.04 ^b^	596.4 ± 0.06 ^a^	6.18	<0.0001	<0.0001	<0.0001	0.0004
NO	15.29 ± 0.75 ^d^	19.75 ± 1.07 ^c^	23.85 ± 0.33 ^b^	27.94 ± 0.46 ^a^	0.71	<0.0001	<0.0001	0.0087	0.9259

^1^ Content: DPPH, 2,2-Diphenyl-1-picrylhydrazyl radical scavenging activity; ABTS, 2,20-Azino-bis(3-ethylbenzothiazoline-6-sulfonic acid) radical scavenging activity; HO, hydroxyl radical scavenging activity; NO, nitric oxide (NO) inhibition. ^2^ SEM: standard error of the mean. ^3^ Contrast: L, linear; Q, quadratic; C, cubic effect. ^a–d^ Means (*n* = 4) with different superscripts within a row differ significantly (*p* < 0.05).

**Table 4:** Carbohydrate, fatty acid, organic acid, and cyclic alcohol metabolites of olive leaves extracts (OLE) identified using GC-MS.

RT (min)	Compound	Formula	Area (%)	MW(g/mol) ^1^	Class ^2^
5.471	Lactic acid	C_3_H_6_O_3_	0.38	90.080	OA
8.894	Glycerol	C_3_H_8_O_3_	0.72	92.094	FA
16.497	Fructose	C_6_H_12_O_6_	1.44	180.160	CHO
16.599	Fructose	C_6_H_12_O_6_	1.10	180.160	CHO
16.704	Galactose	C_6_H_12_O_6_	0.47	180.156	CHO
16.77	Glucose	C_6_H_12_O_6_	10.42	180.156	CHO
16.981	Glucose	C_6_H_12_O_6_	2.06	180.156	CHO
17.12	Sorbitol	C_6_H_14_O_6_	26.30	182.170	CHO
17.189	Ethyl-alpha-glucopyranoside	C_8_H_16_O_6_	3.57	208.210	CHO
18.411	Palmitic acid	C_16_H_32_O_2_	6.84	256.400	OA
18.778	Myo-inositol	C_6_H_12_O_6_	0.95	180.160	CA
20.309	Stearic acid	C_18_H_36_O_2_	6.04	284.480	OA
23.552	Sucrose	C_12_H_22_O_11_	39.71	342.300	CHO

^1^ MW: molecular weight; ^2^ Class: OA, organic acid; FA, fatty acid; CHO, carbohydrate; CA, cyclic alcohol.

**Table 5:** Effects of 5% olive leaves (OLs) on in vitro gas production and fermentation characteristics at 12 and 24 h.

Parameters ^1^	Treatments		SEM ^2^	*p* Value
	Control	5% OLs		
12 h				
pH	7.11 ^b^	7.28 ^a^	0.02	0.0020
DMD (%)	38.00 ^a^	33.67 ^b^	1.07	0.0459
MGR (OD 550 nm)	0.30	0.26	0.01	0.0843
Ammonia (mg∙dL^−1^)	9.80 ^b^	13.49 ^a^	0.25	<0.0001
Total VFA (mM)	48.73 ^a^	45.54 ^b^	0.45	0.0023
Acetate (mM)	31.54 ^a^	29.28 ^b^	0.19	0.0002
Propionate (mM)	9.72	9.59	0.11	0.4291
Butyrate (mM)	7.47 ^a^	6.67 ^b^	0.19	0.0238
A/P ratio	3.24 ^a^	3.05 ^b^	0.03	0.0022
24 h				
pH	6.61	6.66	0.02	0.3034
DMD (%)	51.92 ^a^	49.17 ^b^	0.69	0.0311
MGR (OD 550 nm)	0.35	0.27	0.03	0.1557
Ammonia (mg∙dL^−1^)	11.69 ^b^	13.02 ^a^	0.19	0.0011
Total VFA (mM)	63.26	62.85	0.16	0.1153
Acetate (mM)	41.75	41.64	0.20	0.6941
Propionate (mM)	13.22 ^a^	12.58 ^b^	0.05	0.0002
Butyrate (mM)	8.28 ^b^	8.63 ^a^	0.10	0.0469
A/P ratio	3.16 ^b^	3.31 ^a^	0.02	0.0029

^1^ Parameters: MGR, microbial growth rate; OD, optical density; DMD, dry matter digestibility; VFA, volatile fatty acid; A/P, acetate to propionate ratio. ^2^ SEM: standard error of the means. ^a,b^ Means (*n* = 3) with different superscripts within a row differ significantly (*p* < 0.05).

**Table 6:** Antimicrobial activities of 5% olive leaves (OLs) fractions.

Paper Disk	Microorganisms Tested			
	*Staphylococcus aureus* ATCC 6538	*Escherichia Coli* ATCC 8739	*Klebsiella Pneumoniae* ATCC 4352	*Pseudomonas aeruginosa* ATCC 10145
Conc. (μg/disc)	50	50	50	50
Clear Zone (mm)	10	8	8.5	8

**Table 7:** Effect of 5% olive leaves (OLs) on the relative quantification of rumen microorganism populations under in vitro ruminal fermentation for 12 and 24 h.

Items	Fermentation Time (h)	Control	5% *Olive leaves*	SEM ^1^	*p*-Value
Absolute abundance ^2^					
Total bacteria	12	3.17 ^a^	2.38 ^b^	0.15	0.0212
	24	4.65 ^a^	2.70 ^b^	0.38	0.0216
Fungi	12	34.63	33.69	10.90	0.9543
	24	5.52 ^a^	0.66 ^b^	0.70	0.0079
Ciliate protozoa	12	1.45	3.82	0.85	0.1184
	24	0.97	1.09	0.43	0.8569
Methanogenic archaea	12	10.15	8.87	0.90	0.3712
	24	16.60 ^a^	5.84 ^b^	0.36	<0.0001
Relative proportion, % total bacteria					
*Fibrobacter succinogenes*	12	10.54	10.98	1.44	0.8313
	24	13.20 ^a^	0.53 ^b^	0.38	<0.0001
*Ruminococcus albus*	12	4.27 ^b^	24.12 ^a^	0.54	<0.0001
	24	5.29 ^a^	2.23 ^b^	0.34	<0.0001
*Ruminococcus flavefaciens*	12	0.86 ^b^	1.02 ^a^	0.03	0.0012
	24	0.66	0.63	0.02	0.2469
*Prevotella ruminicola*	12	26.22 ^b^	31.04 ^a^	1.48	0.0349
	24	38.97 ^a^	28.09 ^b^	1.07	<0.0001
*Butyrivibrio fibrisolvens*	12	1.99 ^a^	1.17 ^b^	0.08	0.0012
	24	2.78 ^a^	1.26 ^b^	0.21	0.2469
*Butyrivibrio proteoclasticus*	12	0.36 ^a^	0.26 ^b^	0.01	<0.0001
	24	0.46 ^a^	0.32 ^b^	0.01	<0.0001
*Anearovibrio lipolytica*	12	0.26 ^b^	1.16 ^a^	0.08	<0.0001
	24	0.93 ^b^	4.34 ^a^	0.27	<0.0001

^1^ SEM, standard error of the mean. ^2^ Total bacteria, ×10^10^ copies/mL of ruminal fluid; fungi, ×10^6^ copies/mL of ruminal fluid; ciliate protozoa, ×10^9^ copies/mL of ruminal fluid; methanogenic archaea, ×10^9^ copies/mL of ruminal fluid; ^a,b^ means (*n* = 3) with different superscripts within a row differ significantly (*p* < 0.05).

## Supplementary Materials

The following are available online at https://www.mdpi.com/article/10.3390/ani11072008/s1. Figure S1: Antibacterial activities of olive leaves against Gram-positive and negative bacteria. Gram-positive bacteria: a: *Staphylococcus aureus* (ATCC 6538), Gram-negative bacteria: b: *Escherichia Coli* (ATCC 8739), c: *Klebsiella pneumoniae* (ATCC 4352), d: *Pseudomonas aeruginosa* (ATCC 10145).
