# Supplementary material for: Effects of Olive (Olea europaea L.) Leaves with Antioxidant and Antimicrobial Activities on In Vitro Ruminal Fermentation and Methane Emission
Source: Animals (Basel). 2021 Jul 5;11(7):2008. doi: 10.3390/ani11072008 (PMC8300123; doi:10.3390/ani11072008)
Supplement: Supplementary file 1 [file animals-11-02008-s001.zip › animals-1237964-supplementary.pdf]

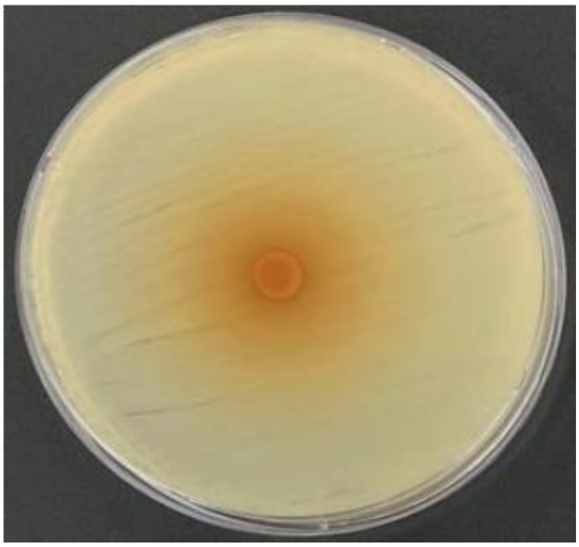

ATCC 4352

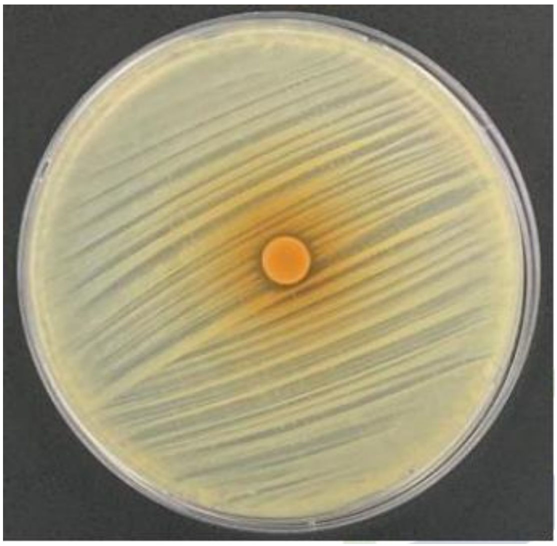

ATCC 6538

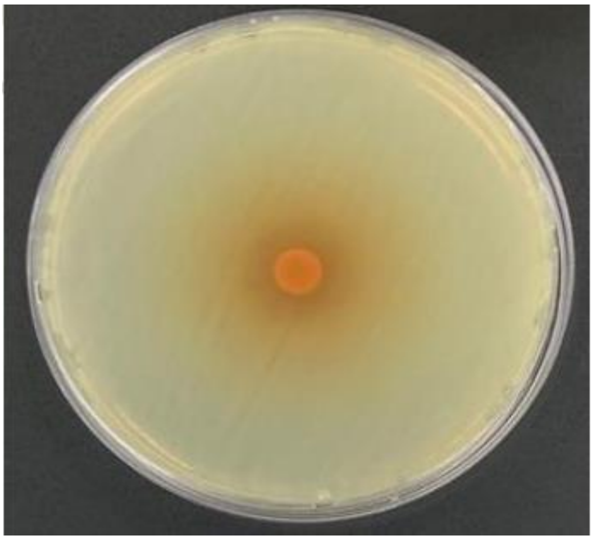

ATCC 8739

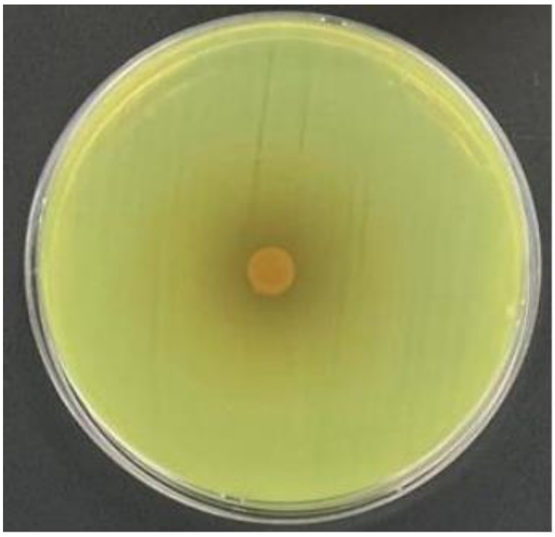

ATCC 10145

Figure S1. Antibacterial activities of Olive leaves against Gram-positive and negative bacteria.
Gram-positive bacteria: a: *Staphylococcus aureus* (ATCC 6538)
Gram-negative bacteria: b: *Escherichia Coli* (ATCC 8739), c: *Klebsiella Pneumoniae* (ATCC 4352), d: *Pseudomonas aeruginosa* (ATCC 10145)
